# Balance between survivin, a key member of the apoptosis inhibitor family, and its specific antibodies determines erosivity in rheumatoid arthritis

**DOI:** 10.1186/ar1498

**Published:** 2005-01-21

**Authors:** Maria Bokarewa, Sofia Lindblad, Dmitriy Bokarew, Andrej Tarkowski

**Affiliations:** 1 Department of Rheumatology and Inflammation Research, Sahlgrenska University Hospital, Göteborg, Sweden

**Keywords:** apoptosis, arthritis, autoimmunity, prognosis, survivin

## Abstract

Rheumatoid arthritis (RA) is a highly heterogeneous disease with respect to its joint destructivity. The reasons underlying this heterogeneity are unknown. Deficient apoptosis in rheumatoid synovial tissue has been recently demonstrated. We have therefore decided to study the synovial expression of survivin, a key member of the apoptosis inhibitor family. The levels of survivin and antibodies against survivin were assessed by an ELISA in matched blood and synovial fluid samples collected from 131 RA patients. Results were related to joint erosivity at the time of sampling. Monocytes were transfected with survivin anti-sense oligonucleotides and were assessed for their ability to produce inflammatory cytokines. Survivin levels were significantly higher in patients with destructive disease as compared with in RA patients displaying a non-erosive disease. High survivin levels were an independent prognostic parameter for erosive RA. In contrast, high levels of antibodies against survivin were found in patients with non-erosive RA, and were negatively related to erosivity. Survivin levels in RA patients were influenced by treatment, being significantly lower among patients treated with disease-modifying anti-rheumatic drugs. Specific suppression of survivin mRNA resulted in downregulation of IL-6 production. We conclude that survivin determines the erosive course of RA, whereas survivin antibodies lead to a less aggressive course of the disease. These findings together with decreased survivin levels upon disease-modifying anti-rheumatic drug treatment, and the downregulation of inflammatory response using survivin anti-sense oligonucleotides, suggest that extracellular survivin expression mediates the erosive course of joint disease whereas autoimmune responses to the same molecule, manifested as survivin targeting antibodies, mediate protection.

## Introduction

Rheumatoid arthritis (RA) is an inflammatory joint disease characterized by hyperplasia of synovial tissue and pannus formation growing invasively into the cartilage, followed by cartilage and bone destruction. Analyses of hyperplastic synovial tissues of patients with RA reveal features of transformed long-living cells such as the presence of somatic mutations, expression of oncogenes, and resistance to apoptosis [1-3]. Resistance to apoptosis further contributes to synovial hyperplasia and is closely linked to the invasive phenotype of synovial fibroblasts [4,5].

Apoptosis is a tightly regulated process of elimination of aged cells without disrupting cellular integrity (reviewed in [6,7]). Apoptosis may be initiated by extracellular stimuli through activation of death receptors on the cell surface, and intracellularly by the release of mitochondrial cytochrome c into the cytoplasm. Both pathways induce expression of apoptosis genes and activation of the caspase cascade, resulting in DNA fragmentation. The apoptosis signals are abrogated by the family of apoptosis-inhibiting proteins (IAPs).

A number of disturbances in the apoptosis machinery have been pointed out in RA patients. Fibroblasts from RA synovia are relatively resistant to apoptosis induced by extracellular Fas stimulation. Moreover, co-culture of synovial fibroblasts from RA joints with T cells and B cells induces anergy of lymphocytes. Increased levels of soluble Fas in RA synovial fluid have been suggested as one possible explanation for this fact [8]. Indeed, administration of antagonistic anti-Fas antibodies or of Fas ligand has been shown effective in abrogation of arthritis in animal models [9,10]. Resistance to Fas-induced apoptosis in RA synovium correlates with a markedly increased expression of sentrin-1 [11]. Sentrin-1/SUMO is a molecule whose binding to a protein results in the prevention of ubiquitin-related processing and degradation of that protein. Sentrin-mediated protection has been shown for such proteins as p53 and IkBa. Upregulation of anti-apoptotic molecules belonging to the Bcl family and of the caspase-8 inhibitor FLIP has been repeatedly reported in RA [12]. Inhibited apoptosis has been shown to contribute to the pathogenesis of experimental arthritis [13,14].

Survivin is a 142-amino-acid protein that belongs to the IAP family, and it inhibits the activity of caspase 3, caspase 7, and caspase 9, but not of the upstream initiator protease caspase 8. Survivin can thereby downregulate, directly or indirectly, both death-receptor-mediated and mitochondria-mediated pathways of apoptosis [15]. Survivin has been also suggested to regulate cell division during mitosis. Indeed, survivin is the only one of IAPs that is tightly connected to the cell cycle being upregulated in the G_2_/M phase. Inside the dividing cell, survivin is found incorporated in centrosomes and mitotic spindles, and relocates to midbodies in the late telophase. Disruption of survivin function by negative mutation or by introduction of anti-sense oligonucleotides results in a cell-division defect [16,17]. Survivin is abundantly expressed in all the most common human cancers and in transformed cell lines [15], while most normal differentiated adult tissues do not express this molecule. A few adult tissues reported to express survivin include the spleen, the testes, the thymi, the placentas, and the colonic crypts.

In the present study we demonstrate high levels of the anti-apoptotic protein survivin extracellularly in plasma and synovial fluid of patients with RA. In all the cases but one, high levels of survivin were associated with the erosive type of joint disease. Moreover, it is demonstrated that autoantibody responses to survivin led to a more benign (non-erosive) course of RA. The latter finding may have potential therapeutic consequences.

## Methods

### Participants

Plasma and synovial fluid samples were collected from 131 RA patients who attended the rheumatology clinics at Sahlgrenska University Hospital, Göteborg for acute joint effusion. RA was diagnosed according to the American College of Rheumatology criteria [18]. At the time of synovial fluid and blood sampling all the patients received non-steroidal anti-inflammatory drugs.

Disease-modifying anti-rheumatic drugs (DMARDs) were used by 96 patients, 67 of which used methotrexate (MTX). Forty-two of these 67 patients combined medication of MTX with the inhibitors of tumour necrosis factor alpha (TNF-α), two other patients combined MTX with sulfasalazine, one patient combined it with cyklosporine A, and the remaining 22 patients were treated with MTX alone. DMARDs other then MTX were used by 14 patients, six patients were treated with sulfasalazine, five patients were treated with cyklosporine A (one patient in combination with azathioprine, one patient with leflunomide, two with sulfasalazine, and the remaining patient with infliximab), four patients used parenteral or oral gold salt compounds, one patient used leflunomide, and one patient used azathioprine. The inhibitors of TNF-α were used in 47 patients (42 patients in combination with MTX, three patients in combination with azathioprine, one patient in combination with cyklosporine, and the remaining patient in combination with cyclophosphamide). The remaining 35 of 131 patients had no DMARD treatment at the time of blood and synovial fluid sampling.

Recent radiographs of the hand and foot skeletons for all patients were studied. The presence of bone erosions, defined as the loss of cortical definition at the joint, was recorded in proximal interphalangeal joints, metacarpophalangeal joints, carpus joints, wrist joints, and metatarsophalangeal joints. The presence of one erosion was sufficient to fulfil the requirement of an erosive disease. We considered the presence of rheumatoid factor (RF) of any of the immunoglobulin isotypes as positive. Informed consent was obtained from the patients and the controls. The study was approved by the Ethics Committee of Sahlgrenska University Hospital.

### Analyses of survivin and antibodies to survivin

Synovial fluid samples were obtained by arthrocentesis of knee joints. Synovial fluid was aspirated aseptically and transferred into tubes containing sodium citrate (0.129 mol/l; pH 7.4). We obtained blood samples simultaneously from the cubital vein and directly transferred them into sodium citrate medium. Blood samples from healthy individuals (*n *= 34; age range, 18–62 years; mean age, 42 ± 7 years) were used as controls. Collected blood and synovial fluid samples were centrifuged at 800 × *g *for 15 min, aliquoted, and stored frozen at -20°C until use.

Survivin levels were determined by a sandwich ELISA using a pair of matched antibodies (rabbit anti-human survivin; R&D Systems, Stockholm, Sweden). Briefly, 96-well polystyrene dishes (Nunc, Roskilde, Denmark) were coated with capture antibodies and were left overnight at room temperature. Following washing, plates were blocked with PBS–BSA containing 5% sucrose. Matched samples of plasma and synovial fluid were introduced into the parallel strips, at a dilution of one in 10 in PBS–BSA. Horseradish peroxidase-labelled detection antibodies and the corresponding substrate were used for colour development. Double-wave reading at 450 and 570 nm was used and the difference of absorbances was calculated. The obtained absorbance values were compared with the serial dilution of recombinant survivin and are presented as picograms per millitre.

Antibodies of IgG and IgM class specific for survivin were measured in blood and synovial fluid samples by an ELISA. Briefly, 96-well polystyrene dishes (Nunc) were coated with human recombinant survivin (R&D Systems). Reconstituted survivin (0.5 μμg/ml) was introduced in each well and left overnight at room temperature. Following washing with PBS containing 0.1% Tween-20, plates were blocked with 1% ovalbumin (Sigma, St Louis, MO, USA) in PBS for 2 hours at room temperature. Matched samples of plasma and synovial fluid were introduced into the parallel strips, in a dilution of one in 100 using PBS–1% ovalbumin. This dilution was established as being on a linear scale in preliminary titration experiments. Horseradish peroxidase-labelled detection antibodies (rabbit F(ab')2-anti-human IgG and IgM; Dako, Glostrup, Denmark), ExtrAvidin peroxidase conjugate (Sigma), and the corresponding substrate were used for colour development. The absorbance at 405 nm was registered. Absorbances of the patient samples were compared with the mean values obtained in the control group of healthy individuals.

### Interaction with survivin transcription

Peripheral blood mononuclear cells (PBMC) were prepared from heparinized blood of healthy individuals by separation on a Lymphoprep density gradient. We washed the cells, and resuspended in complete medium (Iscoves medium containing 1% l-glutamine, 5 × 10^-5 ^M β-mercaptoethanol, 50 μg/ml gentamycin sulphate, and 10% heat-inactivated FCS). We cultured PBMC in 24-well plates in a humidified atmosphere of 5% CO_2 _at 37°C. In addition, we cultured the human monocytic cell line THP-1 (American Type Culture Collection, Manassas, VA, USA) in 10-ml culture flasks (Nunc) in RPMI 1649 medium supplemented with 10% FCS, 1% sodium pyruvate, gentamycin, and 2.5% Hepes in a humidified atmosphere of 5% CO_2 _at 37°C. For the experiments, 4-day-old THP-1 cells were harvested, washed, and adjusted to 1 × 10^6 ^cells/ml.

For the transfection experiments, phosphorothioated oligonucleotides containing the anti-sense-targeting human survivin gene [19] were synthesized by MWG Oligo (Ebersberg, Germany). The following anti-sense sequences were used: aSur 1, 5'-CCCAGCCTTCCAGCTCCTTG-3' ; and aSur 2, 5'-GCACCTAGTCTCCCTGCACC-3'. Irrelevant non-sense sequences were used as controls: non-sense 1, 5'-GTCCTCCACTGGCCTCACTC-3' ; and non-sense 2, 5'-CCCCGATTCACCTCGTCCGT-3'. Oligonucleotides were delivered to THP-1 cells using oligofectamine reagent (Invitrogen, Carlsbad, CA, USA). Before the transfection procedure we seeded THP-1 cells in 96-well tissue culture plates and cultured them overnight in RPMI medium free of antibiotics and FCS. Transfection was performed in RPMI medium supplemented with 2.5% Hepes and 100 mg/ml CaCl_2_. We mixed 0.6 μl oligofectamine with diluted oligonucletides and added it to the washed THP-1 cells. Following 4 hours of incubation at 37°C in a CO_2 _incubator, the transfection procedure was discontinued by adding RPMI medium containing a threefold excess of FCS. At this time point, we also stimulated the cells with phytohaemagglutinine (PHA) (1.5 μg/ml) if required. Following 48 hours of stimulation, THP-1 cultures were aseptically collected, centrifuged at 1000 × *g *for 5 min, and kept frozen at -20°C until analysis. We prepared cell lysates by incubating the cell pellet for 1 hour in 1 mM EDTA buffer containing 6 M urea and proteinase inhibitors (Complete MiniTab; Boehringers, Ingelheim, Germany). These preparations were assessed for proliferation, survivin expression, and IL-6 levels.

Cell survival and apoptosis in the transfected cultures were assessed by surface expression of annexin V and propidium iodide intake. Following transfection and stimulation for 48 hours, THP-1 cells were washed and stained with FITC-marked anti-annexin V antibodies and were subjected to flow cytometry (FACSort; Becton Dickinson, San Jose, CA, USA). The results were analysed using the CELLQuest software (Becton Dickinson).

Proliferation of THP-1 cells was assessed by incubating the cell suspension with the test substance for 48 hours. The cells were then pulsed for 12 hours with 1 μCi [^3^H]thymidine (specific activity, 42 Ci/mmol; Amersham, Bucks, UK). Cells were collected onto glass fibre filters. Thymidine incorporation was measured in a beta-counter. We compared the counts obtained in cells transfected with survivin anti-sense oligonucleotides and those incubated with oligofectamine alone. The results were expressed as a percentage.

The level of IL-6 in supernatants was assessed by a bioassay. The effect of test samples on proliferation of the IL-6-dependent cell line B13.29 [20] was assessed following 72 hours of culturing. The results were analysed by incorporation of [^3^H]thymidine (Amersham) during the last 4 hours of incubation at 37°C. Cells were collected onto a glass fibre filter. Proliferation in the presence of test samples was compared with that induced by standard dilutions of recombinant IL-6 (Genzyme, Cambridge, MA, USA). The results were further recalculated as in the proliferation assay.

### Statistical analysis

We expressed the level of survivin and antibodies against survivin in the blood, in synovial fluid samples, as well as in cell lysates as the mean ± standard error of the mean. The survivin levels in the matched blood and synovial fluid samples were analysed by the paired Student *t *test. We further performed a comparison of survivin levels between the patient blood samples and the healthy controls using the Mann–Whitney U test.

We stratified the patient material according to radiological findings (erosive RA versus non-erosive RA) and calculated the difference in survivin levels between the groups employing the Mann–Whitney U test. An arbitrary level of survivin corresponding to three standard deviations of the control group (300 pg/ml) was chosen as a cut-off. The RA patients were further stratified as having 'high' (>300 pg/ml) or 'low' (<300 pg/ml) levels of survivin. We performed the evaluation of survivin as a prognostic factor for the development of joint destruction, comparing the group having 'high' and 'low' survivin levels in a multivariate analysis. In order to control for the role of other prognostic factors (RF, disease duration, age, presence of antibodies against survivin), a multivariate logistic regression was performed. Odds ratios (with 95% confidence interval) are given for descriptive purposes. All tests were two-tailed and conducted at the 5% significance level.

We evaluated a possible influence of the ongoing treatment on the survivin levels, and we stratified patient material according to DMARD treatment (treated versus untreated). For the simultaneous comparison of the survivin levels in more than two groups the equality of variance *F *test was employed. The inter-relation between the survivin levels and duration of the joint disease, age, white blood cell (WBC) count, and C-reactive protein was calculated employing the Spearman correlation coefficient. For all the statistical evaluation of the results, *P *< 0.05 was considered significant. All statistical evaluations were performed using StatView PowerPC software.

## Results

Clinical and demographic data of the patient population and the control group are presented in Table 1. The patient group showed no difference regarding gender compared with controls, while individuals from the control group were younger (*P *< 0.05). After stratification of the RA patients with respect to radiological changes, the group with erosive joint disease (ERA) was, as expected, more often positive for RF compared with the group for non-erosive joint disease (NRA) (91% versus 23%, *P *< 0.0001), and had longer duration of RA (*P *= 0.0002) as compared with NRA patients. With respect to treatment, 68% of ERA patients were treated with MTX, and 48% in combination with TNF-α inhibitors. Among NRA patients, only 28% were treated with MTX (*P *< 0.025), and 12% with TNF-α inhibitors. NRA patients were significantly more often without DMARDs at the time of blood sampling compared with ERA patients (63% versus 20%, *P *< 0.0001).

### Extracellular survivin determines the erosive course of RA

**Figure 1:**
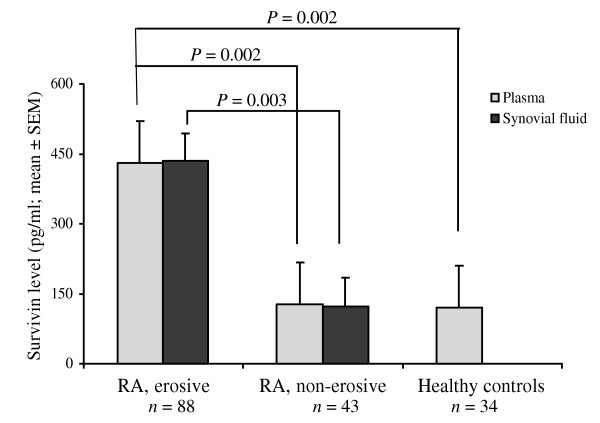
Survivin levels in plasma and synovial fluid of patients with rheumatoid arthritis (RA) are significantly increased in the case of erosive joint disease. SEM, standard error of the mean.

Plasma of the RA patients contained significantly higher levels of survivin as compared with the controls (330 ± 123 pg/ml versus 121 ± 2 pg/ml, *P *= 0.002). Survivin levels in plasma correlated strongly to their levels in synovial fluid (*r *= 0.89). Evaluation of the survivin level was performed in RA patients with respect to the erosivity of joint disease (Fig. 1). Patients with ERA had a significantly higher level of survivin compared with NRA patients in plasma (430 ± 108 pg/ml versus 127 ± 5 pg/ml, *P *= 0.0022) and in the synovial fluid (434 ± 181 pg/ml versus 124 ± 2 pg/ml, *P *= 0.0029). The levels of survivin did not differ significantly between the patients positive for RF (*n *= 90) and those who were RF-negative (*n *= 41) (418 ± 107 pg/ml versus 151 ± 20 pg/ml, not significant). Survivin levels showed no significant correlation to the serum levels of C-reactive protein and WBC count, and neither to the synovial fluid leukocyte count and IL-6 levels.

The RA patients were further stratified as having 'high' (>300 pg/ml) or 'low' (<300 pg/ml) levels of survivin, departing from the level of survivin that corresponded to a mean + three standard deviations of the control group as a cut-off. The difference in the mean survivin level between the 'high' and the 'low' groups was about 10-fold (1180 ± 309 pg/ml versus 97 ± 9 pg/ml). High levels of survivin were detected in 28 of 131 patients (21%). All but one (96%) of the patients with a high survivin level displayed erosive RA. A dominance of a high survivin level among the ERA patients was consequently found both in plasma and in synovial fluid samples. Comparison between the ERA patients having high and low levels of survivin (Table 2) revealed, beside erosivity, an association between high levels of survivin and increased circulating C-reactive protein as well as elevated WBC counts. In contrast, age, gender, RF-positivity, and duration of the disease were similar in the ERA patients with high levels of survivin as compared with those with low levels.

The level of survivin was also studied in RA synovial fluid samples separated with respect to the cell pellet and the supernatant by centrifugation (*n *= 9). Survivin levels found in supernatants and in the lysates of synovial fluid cells obtained from the same sample revealed a strong correlation (*r *= 0.87, *P *< 0.0001). These data indicate that survivin is produced and secreted locally in the joints of RA patients.

To evaluate the predictive value of high survivin levels for the development of destructive joint disease, a logistic regression model was constructed, taking erosive changes at radiological examination of the hand and foot skeletons as a dependent variable. We found that high levels of survivin were significantly associated with erosive changes (odds ratio, 18.76; 95% confidence interval, 2.45–143.65; *P *= 0.0048). To determine whether survivin was independently associated with erosive RA, we developed a multivariate logistic regression model with radiological changes as the dependent variable and with RF, duration of RA, gender, and the survivin level as independent variables. After adjusting for the presence of RF, gender, and the duration of RA, a high level of survivin was significantly associated with erosive RA (odds ratio, 16.02; 95% confidence interval, 2.02–127.19; *P *= 0.013). Our data thus demonstrate that RA patients having high levels of survivin are 16 times more likely to develop erosive joint disease compared with those with low levels of survivin.

**Figure 2:**
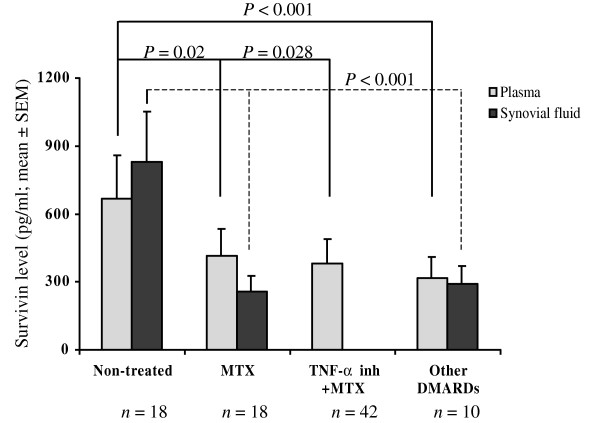
Influence of disease-modifying anti-rheumatic drugs on survivin levels of rheumatoid arthritis patients with erosive joint disease. DMARDs, disease modifying anti-rheumatic drugs; MTX, methotrexate; TNF-α inh, tumour necrosis factor alpha inhibitors; SEM, standard error of the mean.

Taking into account the fact that the increased survivin levels were observed predominantly among the ERA patients, we assessed the effect of DMARD treatment on survivin levels in this patient group. To analyse the putative influence of anti-rheumatic treatment on the level of survivin, ERA patients were stratified with respect to their treatment modality at the time of sampling into three groups. Group 1 included patients receiving MTX (*n *= 18), group 2 included patients treated with combination of MTX and TNF-α inhibitors (*n *= 42), group 3 included patients treated with DMARDs other than MTX (*n *= 10), and group 4 included patients having no treatment with DMARD at the time of sampling (*n *= 18) (Fig. 2). The highest level of survivin, both in blood and in synovial fluid, was found in the group of patients having no DMARD at the time of sampling (blood, 666 ± 473 pg/ml and synovial fluid, 830 ± 610 pg/ml, respectively). This was significantly higher than in the patients treated with MTX (322 ± 174 pg/ml, *P *= 0.02) and in the patients treated with other DMARDs (280 ± 82 pg/ml, *P *< 0.001). These three groups of patients were similar with respect to the duration of the disease, age, WBC counts in blood and synovial fluid, and levels of C-reactive protein. Patients treated with combination of MTX and TNF-α inhibitors exhibited no significant difference in survivin plasma levels compared with the patients treated with MTX alone. This was despite the fact that patients obtaining TNF-α inhibitors were younger (*P *< 0.05) and had lower levels of WBC and C-reactive protein (*P *< 0.05).

### Autoantibodies specific for survivin relate to the non-erosive course of RA

**Figure 3:**
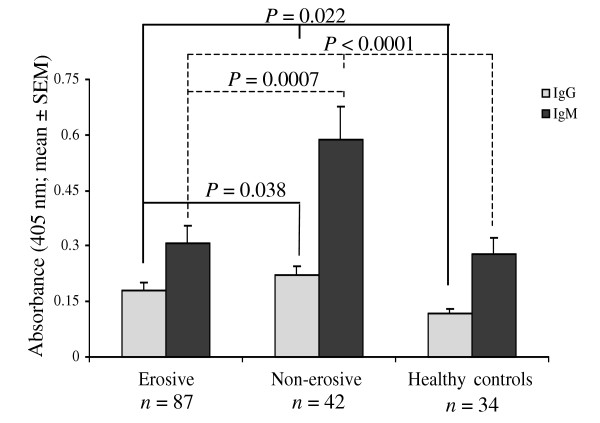
Synovial fluid antibodies of both IgG and IgM isotypes specific for survivin are higher in rheumatoid arthritis patients with the non-erosive course compared with the erosive course of the joint disease. SEM, standard error of the mean.

An ELISA was used for the evaluation of antibodies against survivin of IgG and IgM isotypes in plasma and in synovial fluid of 129 patients with RA and of 34 healthy controls. The absorbance values revealed a significantly higher antibody reactivity with human recombinant survivin in the case of RA patients compared with the controls (Fig. 3). This was true both for IgG (0.19 ± 0.02 versus 0.11 ± 0.012, *P *= 0.022) and for IgM (0.60 ± 0.03 versus 0.28 ± 0.03, *P *< 0.0001) isotypes of antibodies. There was a weak, although significant, correlation between the antibodies of IgG and IgM isotypes in blood (*r *= 0.389, *P *< 0.001), but not in synovial fluid (*r *= 0.146, not significant). No significant difference in the IgG antibody levels was found between blood and synovial fluid (0.19 ± 0.02 versus 0.20 ± 0.03, not significant), while the level of IgM antibodies was significantly higher in blood samples than in synovial fluid samples (0.60 ± 0.03 versus 0.43 ± 0.03, *P *= 0.031).

**Figure 4:**
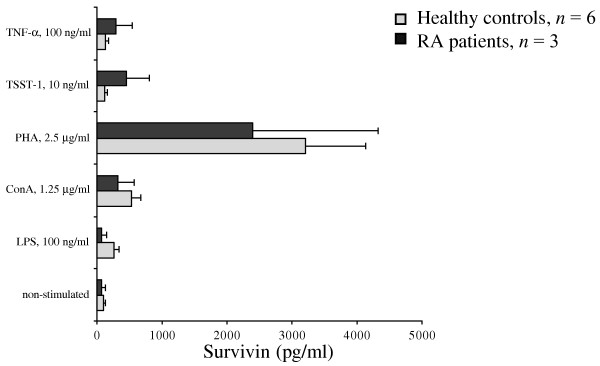
Expression of survivin in lysates from peripheral blood mononuclear cells of rheumatoid arthritis (RA) patients and from healthy controls following stimulation with various mitogens. Survivin expression was measured following 48 hours of stimulation. TNF-α, tumour necrosis factor alpha; PHA, phytohaemagglutinine; ConA, Concanavalin A; LPS, lipopolysaccharide.

Stratification of the patient material with respect to radiological changes revealed that the level of antibodies against survivin was higher in NRA patients compared with ERA patients (Fig. 4). The difference was most pronounced in synovial fluid samples (IgG, 0.18 ± 0.02 versus 0.22 ± 0.02, *P *= 0.038; IgM, 0.31 ± 0.03 versus 0.59 ± 0.03, *P *= 0.0007). Among the ERA patients, a distinct group of patients with high extracellular levels of survivin was outlined. These patients had significantly higher levels of antibodies against survivin both in blood (IgG, 0.25 ± 0.02 versus 0.15 ± 0.02, *P *< 0.0001; IgM, 0.64 ± 0.03 versus 0.55 ± 0.03, not significant) and in synovial fluid (IgG, 0.21 ± 0.02 versus 0.16 ± 0.02, not significant; IgM, 0.40 ± 0.03 versus 0.27 ± 0.03, *P *= 0.023) as compared with those ERA patients with low survivin levels. However, no significant correlation between the level of extracellular survivin and the level of antibodies against survivin was observed (*r *= 0.05).

### Influence of survivin expression on inflammatory responses

PBMC from healthy individuals and from RA patients were stimulated with various B-cell and T-cell mitogens, superantigen, and TNF-α (10–100 ng/ml lipopolysaccharide, 0.5–5 μg/ml Concanavalin A, 10–100 ng/ml TNF-α, 10–100 ng/ml TSST-1, 0.5–5 μg/ml PHA) for 6–48 hours. Supernatants and cell lysates were evaluated for survivin expression by an ELISA. Detectable levels of survivin were not found in supernatants. In the cell lysates, levels of survivin varied in response to the aforementioned stimuli (Fig. 4). In the tested panel, the T-cell mitogen PHA was found to be a potent inducer of survivin expression both by PBMC originating from RA patients (*n *= 3) and by PBMC from healthy controls (*n *= 6)

Stimulation of THP-1 with PHA was therefore used in the subsequent transfection experiments. To assess the role of survivin in the inflammatory process, the human mononuclear cell line THP-1 was transfected with oligonucleotides targeting different regions of survivin mRNA. Oligonucleotides were delivered in complex with oligofectamine as described in Materials and methods. Successful transfection with the inhibitory sequence was confirmed by a downregulation of survivin expression in THP-1 lysates as assessed by ELISA. THP-1 cells displayed, as expected, high spontaneous intracellular expression of survivin, which correlated well with their proliferative activity.

**Figure 5:**
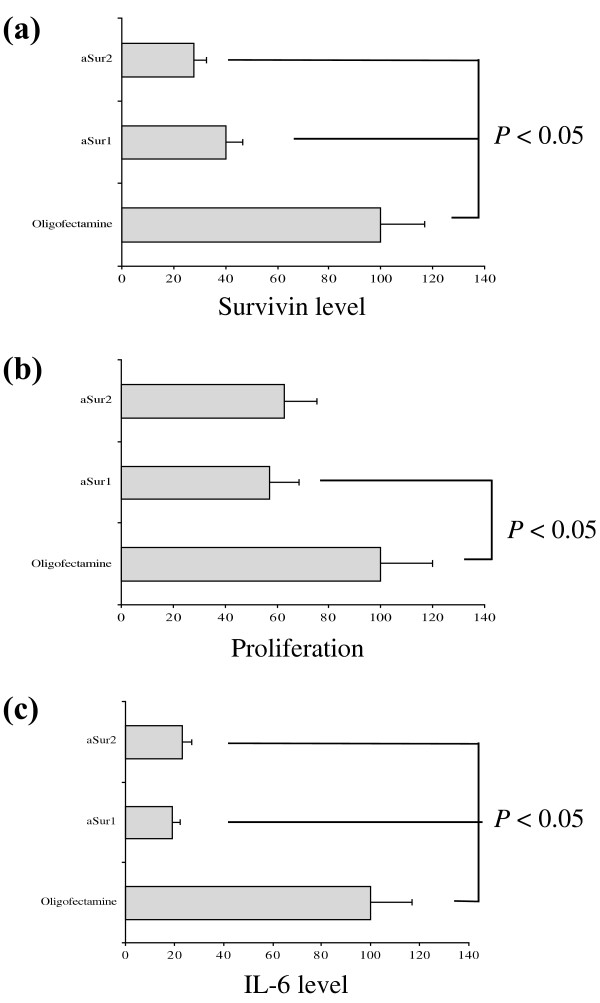
Modulation of **(a) **survivin expression, **(b) **proliferation, and **(c) **IL-6 production following transfection of THP-1 cells with anti-sense oligonucleotides specific for survivin mRNA and non-sense sequences. Data are provided as the percentage of phytohaemagglutinine-stimulated THP-1 cells. The concentration of oligonucleotides throughout was 300 nM.

Following the transfection procedure, cells were stimulated with PHA (1.5 μg/ml) for 48 hours and the cultures were assessed for proliferation and secretion of IL-6. Two different anti-sense sequences were tested, and both anti-sense oligonucleotides downregulated survivin expression (from 100% to 30–44%, *P *< 0.05). In contrast, non-sense oligonucleotides showed no significant suppression of survivin expression as compared with the THP-1 cultures incubated with oligofectamine alone (Fig. 5a). In the THP-1 cultures displaying suppressed survivin expression, a significant downregulation (*P *< 0.01) of IL-6 production was observed, decreasing from 100% to 21–30% (Fig. 5c). To assess whether low survivin expression was related to apoptosis and cell death in the transfected cell cultures, cell proliferation and the expression of annexin V were measured using FACS analysis. THP-1 cells transfected with anti-sense oligonucleotides showed no significant difference regarding annexin V expression (24–37% versus 20–27%, not significant) or proliferation rate (57–68% versus 64–80%, not significant) (Fig. 5b) compared with the cells transfected with non-sense oligonucleotides. These data indicate that the production of inflammatory cytokine IL-6 participating in the regulation of inflammatory responses is directly related to survivin expression by monocytes.

## Discussion

Suppression of apoptosis has been suggested as a key mechanism supporting selection and accumulation of distinct lymphocyte subsets in chronically inflamed joint tissues [21]. Indeed, synovial T cells in RA are highly differentiated and would not normally be expected to survive for a prolonged time within inflamed joints unless their death was actively inhibited [22]. In the present study we demonstrate that high expression of survivin, a member of the IAP family, is a new and potentially important mechanism of apoptosis suppression in patients with RA. Survivin is known as a multipotent inhibitor of apoptosis, neutralizing several caspases at the final steps of the apoptosis cascade, thus abrogating signals from both the death-receptor-dependent and mitochondrial pathways of apoptosis. Together with previous findings of upregulation of other caspase inhibitors (Bcl and FLIP) [12,13], high levels of survivin give new insights in numerous alterations of the apoptosis machinery during the course of RA.

We observed that survivin levels were clearly increased in synovial fluid and plasma of RA patients compared with the healthy controls. Survivin expression was originally considered a reflection of cell proliferation. Indeed, survivin is continuously overexpressed in cancer cells [23]. Survivin gene transcription is repressed by wild-type p53 [24-26]. Multiple mutations and functional dysregulation of p53 have been demonstrated in the synovial tissue of RA patients [3,27] and constitute one of the possible reasons for increased survivin production in this non-malignant condition. Notably, high survivin levels (over three standard deviations of the mean of healthy blood donors) were registered exclusively in patients with erosive joint disease and were associated with markers of inflammation such as WBC count and C-reactive protein levels, as well as with the absence of immunosuppressive treatment. This category of RA patients typically displays chronic joint inflammation, progressive joint destruction, and early mortality [28,29].

Altogether these findings place survivin at the centre of attention as a potential prognostic factor for the destructive course of disease in RA. Indeed, using logistic regression analysis, we demonstrated that RA patients having high levels of survivin had a 16 times higher risk to develop destructive joint disease as compared with the patients with low levels of survivin. Moreover, in a multivariant model we showed that the role of survivin is independent of the presence of RF, the duration of the rheumatic disease, and gender. Interestingly, survivin expression has been shown to be an important prognostic factor in acute leukaemia [30,31], and a predictor of recurrence in soft-tissue sarcomas [32] and urinary bladder cancer [33,34]. In the latter case, extracellular urinary survivin levels were used for the evaluation of treatment and recurrence of cell carcinoma.

Survivin expression determined locally in the inflamed joints and also systemically in circulation of patients with RA was measured extracellularly. Whether survivin found extracellularly originates from dead cells or is a subject of active secretion is presently unknown. The number of *in vitro *leukocyte-activating stimuli (e.g. lipopolysaccharide, PHA, TSST-1, Concanavalin A) will not induce secretion of survivin. This observation suggests, but does not prove, that extracellular survivin found in synovial fluid originates from dead cells. Alternatively, some other cells (e.g. fibroblasts) or endogenous stimuli give rise to secretion of this molecule. Little is known about extracellular functions of survivin. Survivin has been suggested to function as a self-antigen in patients with haematologic malignancies and solid tumours. In our patient material we demonstrate the presence of antibodies to survivin in the plasma and synovial fluid of patients with RA. Interestingly, reactivity against survivin was significantly higher in the patient group with non-erosive RA. Notably, patients with non-erosive RA have extracellular survivin levels undistinguishable from these of the healthy controls. The association of a high level of antibodies against survivin with non-erosive joint disease may be a reflection of a protective autoimmune mechanism existing in these patients.

To assess the role of survivin in the inflammatory process, we first studied its inducibility in differentiated mature human PBMC. Most of the pro-inflammatory stimuli including lipopolysaccharide, Concanavalin A, TSST-1, and TNF-α leading to a significant release of inflammatory cytokines and chemokines, failed to induce survivin expression by PBMC. In contrast, downregulation of survivin expression using specific anti-sense oligonucleotides resulted in the decrease of IL-6 production by human monocytes. These two observations suggest that the regulatory role of survivin in inflammation is mediated by an increase of cytokine production. The connection between survivin expression and production of IL-6 deserves special attention in the view of recent success of the neutralization of IL-6 for alleviation of RA [35]. These observations support the regulatory role of survivin in the pathogenesis of arthritis.

Studying the variability of survivin levels in patients with RA, we observed that in most cases survivin levels were inclined to decrease in survivin-positive patients and almost never converted from absent to high in survivin-negative cases (data not shown). We also showed that the decrease of survivin levels could be mediated by treatment with DMARDs. This suggests survivin to be a transient phenomenon in the course of RA and may explain a relatively low frequency of patients having high survivin levels (21%) in the cohort tested. However, the results of our study may be affected by the fact that most of the patients were treated with DMARDs at the time of sampling, and even those without ongoing DMARD therapy might have received immunosuppressive treatment previously.

## Conclusions

Our study suggests that survivin regulates the inflammatory and destructive process inside the joints of patients with RA. Indeed, high levels of extracellular survivin are associated with chronic erosive arthritis, indicating poor prognosis. In contrast, antibodies against survivin are characteristic of the patients with the non-erosive, benign course of RA. Our findings on survivin expression and autoimmunity to this molecule provide new insight regarding the role of apoptosis in RA.

## Abbreviations

BSA = bovine serum albumin; DMARD = disease-modifying anti-rheumatic drug; ELISA = enzyme-linked immunosorbent assay; ERA = erosive rheumatoid arthritis group; FACS = fluorescence-activated cell sorting; FCS = foetal calf serum; FITC = fluorescein isothiocyanate; IAP = inhibitor of apoptosis proteins; IL = interleukin; MTX = methotrexate; NRA = non-erosive rheumatoid arthritis group; PBMC = peripheral blood mononuclear cells; PBS = phosphate-buffered saline; PHA = phytohaemagglutinine; RA = rheumatoid arthritis; RF = rheumatoid factor; TNF-α = tumour necrosis factor alpha; WBC = white blood cell.

## Competing interests

The author(s) declare that they have no competing interests.

## Authors' contributions

MB contributed to the study design, to the clinical, laboratory and statistical evaluation of material from RA patients, and to preparation of the manuscript. SL performed some of the cell experiments. DB performed ELISA assays, bioassays, and some of the transfection experiments. AT contributed to the conception of the study and the study design, to statistical evaluation of the results, and to preparation of the manuscript.

## Figures and Tables

**Table 1:** Clinical and demographic characteristics of patients with rheumatoid arthritis (RA) and of healthy controls

	Erosive RA (*n *= 88)	Non-erosive RA (*n *= 43)	Controls (*n *= 34)
Age (years) [mean ± standard deviation (range)]	63 ± 2 (28–85)	53 ± 3 (19–83)	42 ± 1.8 (18–67)
Sex (male/female)	26/62	12/31	12/22
Duration of the disease (years)	12.7 ± 1.2	8 ± 1.4	-
Rheumatoid factor (+/-)	80/8	10/33	n.a.
Treatment with DMARDs			
Methotrexate (*n *= 25)	18	7	-
Other DMARDs (*n *= 13)	9	4	
TNF-α blockade (*n *= 47)	42 (37*)	5 (5*)	
None (*n *= 45)	18	27	

n.a., not assesed; DMARD, disease modifying anti-rheumatic drug; TNF-α, tumour necrosis factor alpha. *In combination with methotrexate.

**Table 2:** Clinical comparison of patients with rheumatoid arthritis (RA) expressing high* and low levels of survivin

	Survivin high, erosive RA (*n *= 27)	*P*	Survivin low, erosive RA (*n *= 61)	*P*	Survivin low, non-erosive RA (*n *= 42)^†^
Survivin levels (pg/ml)					
Blood	1180 ± 309	<0.0001	97 ± 9	0.013	127 ± 5
Synovial fluid	1039 ± 523	0.016	132 ± 4	n.s.	124 ± 2
Disease duration (years)	15.5 ± 2.4	n.s.	13.6 ± 1.2	0.0002	8.3 ± 1.4
Age (years)	58 ± 3	n.s.	60 ± 2	0.05	53 ± 3
Rheumatoid factor-positive (*n*)	25	n.s.	53	<0.0001	10
C-reactive protein (mg/l)	45 ± 9	0.035	29 ± 5	n.s.	39 ± 7
White blood cell count (× 10^9^/ml)					
Blood	8.7 ± 0.6	0.038	7.2 ± 0.3	n.s.	7.1 ± 0.3
Synovial fluid	10.8 ± 1.9	n.s.	11.2 ± 2.9	n.s.	13.1 ± 2.8

Continuous parameters are presented as the mean ± standard error of the mean. n.s., not significant. *Level of survivin above 300 pg/ml was considered 'high'. ^†^One patient having a high survivin level is excluded.

## References

[B1] Chou CT, Yang JS, Lee MR (2001). Apoptosis in rheumatoid arthritis – expression of Fas, Fas-L, p53, and Bcl-2 in rheumatoid synovial tissues. J Pathol.

[B2] Tak PP, Zvaifler NJ, Green DR, Firestein GS (2000). Rheumatoid arthritis and p53: how oxidative stress might alter the course of inflammatory diseases. Immunol Today.

[B3] Yamanishi Y, Boyle DL, Rosengren S, Green DR, Zvaifler NJ, Firestein GS (2002). Regional analysis of p53 mutations in rheumatoid arthritis synovium. Proc Natl Acad Sci USA.

[B4] Baier A, Meineckel I, Gay S, Pap T (2003). Apoptosis in rheumatoid arthritis. Curr Opin Rheumatol.

[B5] Firestein GS, Yeo M, Zvaifler NJ (1995). Apoptosis in rheumatoid arthritis synovium. J Clin Invest.

[B6] Kim R, Tanabe K, Uchida Y, Emi M, Inoue H, Toge T (2002). Current status of the molecular mechanisms of anticancer drug-induced apoptosis. The contribution of molecular-level analysis to cancer chemotherapy. Cancer Chemother Pharmacol.

[B7] Hussein MR, Haemel AK, Wood GS (2003). Apoptosis and melanoma: molecular mechanisms. J Pathol.

[B8] Hasunuma T, Kayagaki N, Asahara H, Motokawa S, Kobata T, Yagita H, Aono H, Sumida T, Okumura K, Nishioka K (1997). Accumulation of soluble Fas in inflamed joints of patients with rheumatoid arthritis. Arthritis Rheum.

[B9] Matsuno H, Yudoh K, Nakazawa F, Sawai T, Uzuki M, Nishioka K, Yonehara S, Nakayama J, Ohtsuki M, Kimura T (2002). Antirheumatic effects of humanized anti-Fas monoclonal antibody in human rheumatoid arthritis/SCID mouse chimera. J Rheumatol.

[B10] Ogawa Y, Kuwahara H, Kimura T, Tani Y, Yonehara S, Shiraishi A, Ohtsuki M (2001). Therapeutic effect of anti-Fas antibody on a collagen induced arthritis model. J Rheumatol.

[B11] Franz JK, Pap T, Hummel KM, Nawrath M, Aicher WK, Shigeyama Y, Muller-Ladner U, Gay RE, Gay S (2000). Expression of sentrin, a novel antiapoptotic molecule, at sites of synovial invasion in rheumatoid arthritis. Arthritis Rheum.

[B12] Schedel J, Gay RE, Kuenzler P, Seemayer C, Simmen B, Michel BA, Gay S (2002). FLICE inhibitory protein expression in synovial fibroblasts and at sites of cartilage and bone erosion in rheumatoid arthritis. Arthritis Rheum.

[B13] Kurowska M, Rudnicka W, Kontny E, Janicka I, Chorazy M, Kowalczewski J, Ziolkowska M, Ferrari-Lacraz S, Strom TB, Maslinski W (2002). Fibroblast-like synoviocytes from rheumatoid arthritis patients express functional IL-15 receptor complex: endogenous IL-15 in autocrine fashion enhances cell proliferation and expression of Bcl-x(L) and Bcl-2. J Immunol.

[B14] Perlman H, Liu H, Georganas C, Koch AE, Shamiyeh E, Haines GK, Pope RM (2001). Differential expression pattern of the antiapoptotic proteins, Bcl-2 and FLIP, in experimental arthritis. Arthritis Rheum.

[B15] Li F (2003). Survivin study: what is the next wave?. J Cell Physiol.

[B16] Li F, Altieri DC (1999). Transcriptional analysis of human survivin gene expression. Biochem J.

[B17] Uren AG, Wong L, Pakusch M, Fowler KJ, Burrows FJ, Vaux DL, Choo KH (2000). Survivin and the inner centromere protein INCENP show similar cell-cycle localization and gene knockout phenotype. Curr Biol.

[B18] Arnett FC, Edworthy SM, Bloch DA, McShane DJ, Fries JF, Cooper NS, Healey LA, Kaplan SR, Liang MH, Luthra HS (1988). The American Rheumatism Association 1987 revised criteria for the classification of rheumatoid arthritis. Arthritis Rheum.

[B19] Olie RA, Simoes-Wust AP, Baumann B, Leech SH, Fabbro D, Stahel RA, Zangemeister-Wittke U (2000). A novel antisense oligonucleotide targeting survivin expression induces apoptosis and sensitizes lung cancer cells to chemotherapy. Cancer Res.

[B20] Helle M, Boeije L, Aarden LA (1988). Functional discrimination between interleukin 6 and interleukin 1. Eur J Immunol.

[B21] Buckley CD (2003). Why do leucocytes accumulate within chronically inflamed joints?. Rheumatology (Oxford).

[B22] Salmon M, Scheel-Toellner D, Huissoon AP, Pilling D, Shamsadeen N, Hyde H, D'Angeac AD, Bacon PA, Emery P, Akbar AN (1997). Inhibition of T cell apoptosis in the rheumatoid synovium. J Clin Invest.

[B23] Altieri DC (2003). Survivin, versatile modulation of cell division and apoptosis in cancer. Oncogene.

[B24] Hoffman WH, Biade S, Zilfou JT, Chen J, Murphy M (2002). Transcriptional repression of the anti-apoptotic survivin gene by wild type p53. J Biol Chem.

[B25] Mirza A, McGuirk M, Hockenberry TN, Wu Q, Ashar H, Black S, Wen SF, Wang L, Kirschmeier P, Bishop WR (2002). Human survivin is negatively regulated by wild-type p53 and participates in p53-dependent apoptotic pathway. Oncogene.

[B26] Zhou M, Gu L, Li F, Zhu Y, Woods WG, Findley HW (2002). DNA damage induces a novel p53-survivin signaling pathway regulating cell cycle and apoptosis in acute lymphoblastic leukemia cells. J Pharmacol Exp Ther.

[B27] Seemayer CA, Kuchen S, Neidhart M, Kuenzler P, Rihoskova V, Neumann E, Pruschy M, Aicher WK, Muller-Ladner U, Gay RE (2003). p53 in rheumatoid arthritis synovial fibroblasts at sites of invasion. Ann Rheum Dis.

[B28] Krause D, Schleusser B, Herborn G, Rau R (2000). Response to methotrexate treatment is associated with reduced mortality in patients with severe rheumatoid arthritis. Arthritis Rheum.

[B29] Savolainen A, Isomaki H, Myllykangas-Luosujarvi R, Aho K (2000). Trends in mortality of patients with rheumatoid arthritis. J Rheumatol.

[B30] Kamihira S, Yamada Y, Hirakata Y, Tomonaga M, Sugahara K, Hayashi T, Dateki N, Harasawa H, Nakayama K (2001). Aberrant expression of caspase cascade regulatory genes in adult Tcell leukaemia: survivin is an important determinant for prognosis. Br J Haematol.

[B31] Paydas S, Tanriverdi K, Yavuz S, Disel U, Sahin B, Burgut R (2003). Survivin and aven: two distinct antiapoptotic signals in acute leukemias. Ann Oncol.

[B32] Kappler M, Kotzsch M, Bartel F, Fussel S, Lautenschlager C, Schmidt U, Wurl P, Bache M, Schmidt H, Taubert H (2003). Elevated expression level of survivin protein in soft-tissue sarcomas is a strong independent predictor of survival. Clin Cancer Res.

[B33] Smith SD, Wheeler MA, Plescia J, Colberg JW, Weiss RM, Altieri DC (2001). Urine detection of survivin and diagnosis of bladder cancer. JAMA.

[B34] Hausladen DA, Wheeler MA, Altieri DC, Colberg JW, Weiss RM (2003). Effect of intravesical treatment of transitional cell carcinoma with bacillus Calmette-Guerin and mitomycin C on urinary survivin levels and outcome. J Urol.

[B35] Yoshizaki K, Nishimoto N, Mihara M, Kishimoto T (1998). Therapy of rheumatoid arthritis by blocking IL-6 signal transduction with a humanized anti-IL-6 receptor antibody. Springer Semin Immunopathol.

